# A multi-model approach identifies ALW-II-41-27 as a promising therapy for osteoarthritis-associated inflammation and endochondral ossification

**DOI:** 10.1016/j.heliyon.2024.e40871

**Published:** 2024-12-04

**Authors:** Mauricio N. Ferrao Blanco, Raphaelle Lesage, Nicole Kops, Niamh Fahy, Fjodor T. Bekedam, Athina Chavli, Yvonne M. Bastiaansen-Jenniskens, Liesbet Geris, Mark G. Chambers, Andrew A. Pitsillides, Roberto Narcisi, Gerjo J.V.M. van Osch

**Affiliations:** a Department of Orthopaedics and Sports Medicine, Erasmus MC, University Medical Center Rotterdam, Rotterdam, the Netherlands; b Prometheus, Division of Skeletal Tissue Engineering, KU Leuven, Belgium; c Biomechanics Section, KU Leuven, Belgium; d Department of Oral and Maxillofacial Surgery, Erasmus MC, University Medical Center Rotterdam, Rotterdam, the Netherlands; e Department of Applied Science, Technological University of the Shannon: Midlands Midwest, Limerick, Ireland; f GIGA In Silico Medicine, University of Liège, Belgium; g Lilly Research Laboratories, Eli Lilly Pharmaceuticals, Indianapolis, USA; h Comparative Biomedical Sciences, Royal Veterinary College, London, United Kingdom; i Department of Otorhinolaryngology, Erasmus MC, University Medical Center Rotterdam, Rotterdam, the Netherlands; j Department of Biomechanical Engineering, University of Technology Delft, Delft, the Netherlands

**Keywords:** Chondrocyte hypertrophy, Inflammation, Kinase inhibitor, Virtual cell, Drug target, Author e-mails

## Abstract

Low-grade inflammation and pathological endochondral ossification are key processes underlying the progression of osteoarthritis, the most prevalent joint disease worldwide. In this study, we employed a multi-faceted approach, integrating publicly available datasets, *in silico* analyses, *in vitro* experiments and *in vivo* models to identify new therapeutic candidates targeting these processes. Data mining of transcriptomic datasets identified EPHA2, a receptor tyrosine kinase associated with cancer, as being linked to both inflammation and endochondral ossification in osteoarthritis. A computational model of cellular signaling networks in chondrocytes predicted that *in silico* activation of EPHA2 in healthy chondrocytes increases inflammatory mediators and induces hypertrophic differentiation, a hallmark of endochondral ossification. We then evaluated the effect of EPHA2 inhibition using the tyrosine kinase inhibitor ALW-II-41-27 in cultured human chondrocytes from individuals with osteoarthritis, demonstrating significant reductions in both inflammation and hypertrophy. Additionally, systemic subcutaneous administration of ALW-II-41-27 in a mouse osteoarthritic model attenuated joint degeneration by reducing local inflammation and pathological endochondral ossification. Collectively, this study demonstrates a novel drug discovery pipeline that integrates computational, experimental, and animal models, paving the way for the development of disease-modifying treatments for osteoarthritis.

## 1 Introduction

Osteoarthritis (OA) stands as the most widespread and debilitating musculoskeletal condition worldwide, characterized by progressive joint alterations leading to pain and functional limitations [1,2]. In OA, joint inflammation ensues, resulting in the loss of cartilage lining the joint surface and the formation of bony outgrowths known as osteophytes at the joint edges. Current pharmacological treatments mainly focus on symptom management, primarily using pain relievers that do not impede disease advancement. Therefore, there is an imperative need to discover drugs capable of modifying OA progression.

Chondrocyte hypertrophy, a phenotype preceding cartilage calcification and eventual replacement by bone, plays a crucial role in OA pathogenesis. Under normal circumstances, articular chondrocytes resist hypertrophic changes. However, in OA, the onset of hypertrophic differentiation accelerates cartilage breakdown [3]. Similarly, joint margins are established through the growth of an initial cartilage template that undergoes endochondral ossification and is replaced by bone [4]. The inflammatory environment in OA, characterized by synovitis, involves heightened macrophage activation and the release of inflammatory cytokines like tumor necrosis factor-alpha (TNF-α) [5,6]. Activation of inflammatory signaling pathways not only leads to cartilage matrix degradation but also triggers chondrocyte hypertrophy [7,8].

We hypothesize that targeting a key regulator governing both chondrocyte hypertrophy and inflammation could serve as an effective therapeutic approach for OA. In the present study we aim to identify a druggable target for OA associated with chondrocyte hypertrophy and inflammation and determine the efficacy of its pharmacologic inhibition combining *in silico* (computational), *in vitro* and *in vivo* models. Computational modelling has revolutionized drug discovery by enabling more precise identification of therapeutic targets [9]. Systems biology techniques offer valuable tools for organizing and analyzing large datasets, transforming them into actionable insights. By leveraging publicly available transcriptomic data and employing computational modeling, we identified EPHA2 in a more comprehensive and unbiased manner.

EPHA2, a receptor tyrosine kinase, has been implicated in various human diseases such as cancer and inflammatory disorders [[10], [11], [12]]. When EPHA2 binds to its ligands, ephrins, it activates its kinase activity, leading to the phosphorylation of specific tyrosine residues (Y588 and Y594) within its juxtamembrane region [13]. In addition to these ligand-mediated activation, inflammatory cytokines such as TNFα has been shown to activate EPHA2, inducing its phosphorylation at Serine 897 [14]. These phosphorylation events are crucial for activating cellular responses mediated by EPHA2 signaling pathway, including cell growth, migration and differentiation [15].

Subsequent to the *in silico* analyses, *in vitro* and *in vivo* experiments confirmed the predictions of EPHA2 as target for inflammation and chondrocyte hypertrophy and demonstrated the therapeutic potential of the tyrosine kinase inhibitor ALW-II-41-27 that has a high selectivity for EPHA2. This multi-faceted approach provides a strong foundation for future drug development efforts, offering a promising new avenue for the treatment of osteoarthritis.

## 2 Methods

### 2.1 Data mining of microarray datasets

Three previously published datasets were leveraged for the purpose of the current study. To identify a target associated with chondrocyte hypertrophy, we used a dataset previously generated in different zones of the growth plate of 14 days old female Swiss White mice [16], using a 44k whole genome oligo microarrays (G4122A; Agilent Technologies, Santa Clara, CA, United States). A list of differently expressed genes between the proliferative (PR) and hypertrophic (H) layer was obtained from the supplementary data of the publication, generated via paired *t*-test and selected factors with a cut off value of 3-fold change in the expression of genes. To identify a target associated with osteoarthritis, we used a dataset previously generated in 10-week-old male C57BL/6 mice by surgical destabilization of the medial meniscus (DMM) [17] using a 44k whole genome oligo microarrays (G4122A; Agilent Technologies). A list of differently expressed genes from cartilage of DMM- or sham-operated mouse joints was obtained from the supplementary data of the publication, generated via paired *t*-test and selected factors with a cut off value of 2-fold change in the expression of genes. To identify targets associated with human osteoarthritic cartilage we used a microarray dataset previously generated [18], performed using Affymetrix oligonucleotide microarray HG-U133plus2.0 (Affymetrix, Santa Clara, CA). Differently expressed genes in articular cartilage from OA and healthy donors were obtained from the supplementary data of the publication, generated via paired *t*-test and considering genes that displayed a mean fold change greater than 2. An online resource from Gene Ontology (GO) was used to obtain a list of genes associated with inflammation, namely GO: 0006954 Inflammatory response [19,20]. The selected lists of uniquely expressed factors were then overlapped utilizing the Funrich software [21], to select for genes associated with osteoarthritis, inflammation and chondrocyte hypertrophy.

### 2.2 *In silico* experiments (simulations)

A computational model of the intracellular signaling pathways regulating articular chondrocyte phenotypes [22,23], was leveraged and completed with information about EPHA2. This mechanistic model represents 62 molecular entities (growth factors, receptors, transcription factors, extracellular matrix proteins and signaling) and the way they regulate each other, at protein and genetic level, to transduce external signals and define new cellular states (see list of variables in Supplementary Table S2 and the full list of interactions, in the standard.csv format in Supplementary Data 1 or at https://github.com/Rapha-L/Virtual_Chondrocyte_for_EPHA2_study). Connections of EPHA2 with the rest of the signaling network were added according to information found in literature (Fig. 2A and Supplementary Table 1) with supporting references featuring different cell types, given that EPHA2 is still understudied in cartilage. The mathematical implementation consists of additive equations in which each variable (i.e. biomolecule) is regulated at protein (fast) and genetic (slow) level with values continuously ranging between 0 (fully repressed) and 1 (fully expressed and activated). The global activity is defined as the multiplication of the genetic activation level and protein activation level. An asynchronous updating scheme with priority classes (i.e. fast sub-variables updated first) was employed, resulting in semi-quantitative stochastic simulations [24]. In the algorithm executing the asynchronous updating scheme to simulate the model, the fast (protein signaling level) and slow (genetic level) sub-variables are updated one-by-one with two different priority classes. A new slow sub-variable is updated when all fast sub-variables have been updated and when a pseudo-stable state has been reached at the fast (protein signaling) level. The order in which fast and slow sub-variables are updated within a priority class is random (MATLAB random seed via randperm(n) function). The algorithm counts a new computational timestep each time a new slow sub-variable is updated, this is how time-trajectories are obtained. The outcome of a given perturbation may vary, due to the random order in which variables are updated. This reflects actual biochemical processes, in which there is a random component for two molecules to enter in contact and react together/influence each other in the cytoplasm or nucleus, due to inherent entropy. Hence, each perturbation has a certain propensity to trigger a state transition. Therefore, each perturbation is repeated 100 times in order to compute the percentage of repetition triggering a state transition. The only parameter of the model is a saturation constant affecting the weight of a regulatory interaction in the network. The saturation constant was assigned an arbitrary value (s = 2/3) based on a previous study which evaluated the influence of that constant on a similar model [24]. This constant determines how fast a protein activity or gene expression can saturate to the maximal value depending on the amount of excess positive and negative upstream interactions.

A Monte Carlo approach (10,000 random initializations) was used to compute the model's stables states, equating to possible molecular states of the cell (i.e. chondrocyte phenotypes). The stable states were characterized regarding the variable (i.e. biomolecules' activity) as markers. In particular, the states with a high (resp. low) activity for SOX9 and low (resp. high) activity for RUNX2 where considered as healthy (resp. hypertrophic) chondrocytes. *In silico* experiments were initially executed using the model's stable state resembling the most a regular healthy chondrocyte. *In silico* experimental conditions were applied by (simultaneously) forcing the targeted variable(s) to be set to 0 (for inhibition) or 1 (for activation), unless specified otherwise (e.g. variation of EPHA2 activity in Fig. 2B). The *in silico* conditions were applied for 1000 computing steps after which all variables were left free to evolve until reaching a new stable state, thereby simulating a bolus treatment effect. This was repeated 100 times and the various outcomes, or final states, (due to the model stochasticity) were averaged to compute the final profiles, standard deviations were also computed. When a final state is different from the initial state before perturbation, this is called a state transition. The percentage of transition to each emerging final state was also computed over the 100 repetitions. The period during which the perturbation was maintained was set to 1000 timesteps, as it is largely greater than the time typically needed (about 400steps) for all variables to reach a steady state in the perturbed scenarios. The following 4 *in silico* conditions (or perturbations) were applied: (1) activation of EPHA2 (variable #62), (2) activation of pro-inflammatory cytokines (variable #51), (3) the combination of the two previous conditions and (4) blockage of EPHA2 while activating the pro-inflammatory cytokines. The model and associated code are available via the following GitHub repository: https://github.com/Rapha-L/Virtual_Chondrocyte_for_EPHA2_study.

### 2.3 Evaluation of ALW-II-41-27 in OA chondrocytes and cartilage explants

Human articular cartilage was obtained with implicit consent as waste material from patients undergoing total knee replacement surgery. This protocol was approved by the Medical Ethical Committee of the Erasmus MC, University Medical Center, Rotterdam, protocol number MEC-2004-322. Full thickness cartilage explants (ø = 5 mm) were harvested from macroscopically intact areas and washed twice with 0.9 % NaCl (Sigma Aldrich, St. Louis, MO, USA). To isolate chondrocytes, cartilage chips were subjected to protease (2 mg/mL, Sigma Aldrich) for 2 h followed by overnight digestion with 1.5 mg/mL collagenase B (Roche Diagnostics, Basel, Switzerland) in Dulbecco's modified Eagle's medium (DMEM) high glucose supplemented with 10 % fetal bovine serum. Single cell suspension was obtained by filtrating the cellular solution by a 100 μm filter. The isolated chondrocytes were expanded in monolayer at a seeding density of 7500 cells/cm2 in DMEM high glucose supplemented with 10 % fetal bovine serum, 50 μg/mL gentamicin, and 1.5 μg/mL fungizone (Gibco, Grand Island, NY, USA). Approximately 80 % confluency cells were trypsinized and reseeded at 7500 cells/cm2. Cells were used for experiments after 3 passages. To re-differentiate the expanded articular chondrocytes, a 3D alginate bead culture model was used [25,26]. Alginate beads were prepared by mixing passage three chondrocytes and re-suspended them in 1.2 % (w/v) low viscosity alginate (Kelton LV alginate, Kelko Co, San Diego, CA, USA) in 0.9 % NaCl (Sigma Aldrich) at a concentration of 4 × 10^6^ cells/mL. Beads were made by dripping the cell-alginate suspension in 105 mM CaCl_2_ (Sigma Aldrich) through a 22-gauge needle. Beads were washed with 0.9 % NaCl and DMEM low glucose. Beads with a size that deviated from the average after a visual inspection were not included in the experiment. Re-differentiation of chondrocytes was performed in a 24-well plate (BD Falcon) for two weeks in 100 μL/bead DMEM low glucose supplemented with 1 % v/v Insulin-Transferrin-Selenium (ITS™+ Premix, Corning, Bedford, MA, USA), 10 ng/mL transforming growth factor beta-1 (TGF-β-1, recombinant human, R&D systems) 25 μg/mL L-ascorbic acid 2-phosphate (Sigma Aldrich), 50 μg/mL gentamicin, and 1.5 μg/mL fungizone (both Gibco). After two weeks, TGF-β-1 was no longer added to the medium and cells were cultured with 10 μM of ALW-II-41-27 (MedChemExpress, Ann Arbor, MI, USA), vehicle (DMSO) and/or with the pro-inflammatory cytokine TNF-α 10 ng/mL for 24 h. Preliminary experiments were performed using two doses of ALW-II-41-27, 1 and 10 μM, being 10 μM more effective. Medium and alginate beads were harvested for further analyses.

### 2.4 Evaluation of ALW-II-41-27 in chondrogenically differentiated MSCs

Human MSCs were isolated from surplus iliac crest bone chip material harvested from pediatric patients undergoing alveolar bone graft surgery. All human samples were obtained with the approval of the Erasmus MC, University Medical Center Medical Research Ethics Committee (MEC-2014-16). Written consent was not required in accordance with the national code regarding the use of waste surgical material for scientific research (www.coreon.org), and an opt-out option was available. Iliac crest bone chips were washed with expansion medium composed of Minimum Essential Medium (MEM)-α (containing nucleosides) supplemented with heat inactivated 10 % v/v fetal bovine serum (FBS) (both Thermo Fisher Scientific, Waltham, MA, USA), 1.5 μg/mL fungizone (Gibco), 50 μg/mL gentamicin (Gibco), 25 μg/ml L-ascorbic acid 2-phosphate (Sigma-Aldrich, St. Louis, MO, USA), and 1 ng/mL fibroblast growth factor-2 (Instruchemie, Delfzijl, The Netherlands), and the resulting cell suspension was seeded in T75 flasks. Cells were washed twice with phosphate buffered saline (Thermo Fisher Scientific) supplemented with 2 % v/v heat inactivated FBS 24 h following seeding to remove non-adherent cells. MSCs were cultured at 37 °C and 5 % carbon dioxide under humidified conditions, with expansion medium refreshed every 3–4 days. MSCs were sub-cultured upon reaching 80–90 % confluency using 0.25 % w/v trypsin-EDTA (Thermo Fisher Scientific) and reseeded at a cell density of 2300 cells/cm^2^. MSCs were used at passage 3 for chondrogenic pellet cultures.

For chondrogenic differentiation, 2 × 10^5^ MSCs were suspended in 500 μl of chondrogenic differentiation medium composed of high glucose Dulbecco's Modified Eagle Medium supplemented with 1.5 μg/mL fungizone (Gibco), 50 μg/mL gentamicin (Gibco), 1 mM sodium pyruvate (Thermo Fisher Scientific), 1 % v/v Insulin-Transferrin-Selenium (ITS™+ Premix, Corning, Bedford, MA, USA), 40 μg/mL proline (Sigma-Aldrich), 25 μg/ml L-ascorbic acid 2-phosphate (Sigma-Aldrich), 100 nM dexamethasone (Sigma-Aldrich), and 10 ng/mL TGF-β-1 (R&D systems). The cell suspension was added to 15 mL conical polypropylene tubes (TPP, Radnor, PA, USA) and centrifuged at 300 g for 8 min to facilitate pellet formation. Chondrogenic MSC pellets were cultured at 37 °C and 5 % carbon dioxide in a humidified atmosphere. After 24 h, pellets were tapped to enhance pellet formation and the medium was renewed with chondrogenic medium with 100 nM of ALW-II-41-27 (MedChemExpress, Ann Arbor, MI, USA) or vehicle (DMSO). Afterwards the medium was renewed two times per week for a period of 3 weeks.

### 2.5 Nitric oxide (NO) assay

NO production was measured in the medium of OA chondrocytes by determining the content of nitrite using the Griess reagent (Sigma Aldrich). The reaction was monitored at 540 nm using a spectrophotometer (VersaMax; Molecular Devices, Sunnyvale, USA). Sodium nitrite (NaNO_2_; Chemlab, Zedelgem, Belgium) was used as standard for the calibration curve.

### 2.6 Interleukin-6 assay

A commercially available enzyme-linked immunosorbent assay (ELISA) kit was used to determine the concentration of IL-6 in the medium of OA chondrocytes as per manufacturer's instructions (R&D systems, Minneapolis, MN, USA).

### 2.7 Histological analysis of chondrogenic pellets

After 3 weeks of chondrogenic induction, pellets were fixed with 4 % (v/v) formaldehyde in phosphate buffered saline, embedded in paraffin and sectioned (6 μm). Glycosaminoglycan (GAG) was stained with 0.04 % thionine solution and collagen type II was immunostained using a primary antibody II-II6B3 (Developmental Studies Hybridoma Bank, Iowa City, IA, USA) 0.4 μg/mL in PBS/1 % bovine serum albumin (BSA; Sigma-Aldrich), collagen type X using primary antibody 14-9771-82, 5 μg/mL in PBS/1 % BSA (Thermofisher, Waltham, MA, USA). Antigen retrieval for collagen type II was performed with 1 mg/mL pronase (Sigma-Aldrich) in PBS for 30 min at 37 °C, followed by incubation with 1 % hyaluronidase (Sigma-Aldrich) in PBS for 30 min at 37 °C to improve antibody penetration. Antigen retrieval for collagen type X was done by a 2h incubation in 1 mg/mL pepsin in 0.5M acetic acid followed by incubation with 1 % hyaluronidase (Sigma-Aldrich) in PBS for 30 min at 37 °C. The slides were pre-incubated with 10 % normal goat serum (Sigma-Aldrich) in PBS with 1 % BSA (Sigma-Aldrich). Next, the slides were incubated for 1 h (for collagen type II) or overnight (for collagen type X) with the primary antibody, and then with a biotin-conjugated secondary antibody (HK-325-UM, Biogenex, Fremont, CA, USA), alkaline phosphatase-conjugated streptavidin (HK-321-UK, Biogenex), and the Neu Fuchsin chromogen (B467, Chroma Gesellschaft). An IgG1 isotype antibody (X0931, Dako Cytomation, Santa Clara, CA, USA) was used as negative control.

### 2.8 Gene expression

Alginate beads were dissolved using citrate buffer, centrifuged at 200 g and the pellet was resuspended in RLT (Qiagen, Hilden, Germany) buffer containing 1 % beta-mercaptoethanol for RNA isolation. RNA was isolated from the cartilage explants by snap freezing in liquid nitrogen followed by pulverization using a Mikro-Dismembrator (B. Braun Biotech International GmbH, Melsungen, Germany) at 2800 rpm. The tissue was homogenized with 18 μL/mg sample RNA-Bee TM (Tel-Test Inc., Friendswood, TX, USA) and 20 % chloroform.

The MSCs chondrogenic pellets were homogenized in RNA-Bee TM (Tel-Test Inc., Friendswood, USA) and 20 % chloroform was added to extract RNA. mRNA isolation was performed according to manufacturer's protocol utilizing the RNeasy Column system (Qiagen, Hilden, Germany). The RNA concentration was determined using a NanoDrop spectrophotometer (Isogen Life Science, Utrecht, The Netherlands). 0.5 μg RNA was used for cDNA synthesis following the protocol of the manufacturer of the RevertAid First Strand cDNA kit (Thermo Fisher Scientific, Waltham, MA, United States). qPCR was performed on a Bio-Rad CFX96 Real-Time PCR Detection System (Bio-Rad) to assess gene expression, Alkaline phosphatase (*ALPL*, Fw: GACCCTTGACCCCCACAAT; Rev: GCTCGTACTGCATGTCCCCT; Probe: TGGACTACCTATTGGGTCTCTTCGAGCCA), Collagen type 2 (*COL2A1*; Fw: GGCAATAGCAGGTTCACGTACA; Rev: CGATAACAGTCTTGCCCCACTT; Probe: CCGGTATGTTTCGTGCAGCCATCCT), Collagen type 10 (*COL10A1*; Fw: CAAGGCACCATCTCCAGGAA; Rev: AAAGGGTATTTGTGGCAGCATATT; Probe: TCCAGCACGCAGAATCCATCTGA), matrix metalloproteinase-13 (*MMP13*; Fw: AAGGAGCATGGCGACTTCT; Rev: TGGCCCAGGAGGAAAAGC; Probe: CCCTCTGGCCTGCGGCTCA), Runt-related transcription factor 2 (*RUNX2*; Fw: ACGTCCCCGTCCATCCA; Rev: TGGCAGTGTCATCATCTGAAATG; Probe: ACTGGGCTTCTTGCCATCACCGA), Tumor Necrosis Factor-a (*TNFA*; Fw: GCCGCATCGCCGTCTCCTAC; Rev: AGCGCTGAGTCGGTCACCCT). Glyceraldehyde-3-phosphate dehydrogenase (*GAPDH*; Fw: ATGGGGAAGGTGAAGGTCG; Rev: TAAAAGCAGCCCTGGTGACC; Probe: CGCCCAATACGACCAAATCCGTTGAC) was found stable and therefore used as reference gene. Data were analyzed by the ΔΔCt method and normalized to the expression of *GAPDH* of each condition and compared to the corresponding gene expression in the control groups.

### 2.9 Western blot analysis

Mesenchymal stromal cells were expanded to approximately 50 % confluence. Next, medium was refresh with α-MEM supplemented with 1 mg/mL bovine serum albumin (Sigma), 1.5 μg/mL Amphotericin B (Invitrogen), 25 μg/mL L-ascorbic acid 2-phosphate (Sigma-Aldrich), 50 μg/mL gentamycin (Invitrogen). The following day, cells were first exposed to 0,1–10 μM ALW-II-41-27 (Cayman Chemicals) for 1 h and then treated with 10 ng/mL TNF-α and 0,1–10 μM ALW-II-41-27. After 30 min cells were washed with PBS on ice and incubated with mammalian cell lysis buffer, supplemented with 1 % Halt Protease Inhibitor (Thermo Scientific) and 1 % Halt Phosphatase Inhibitor (Thermo Scientific). Cells were manually scraped off the surface and cell lysate was harvested and stored at −20 °C. Samples were sonicated using a BioRuptor® Pico sonication device for 10 cycles of 30 s on and 30 s off. Samples were centrifuged to remove remaining cell debris; the protein concentration was determined using a BCA assay and protein contents were analyzed by Western blot. Proteins were denatured in reducing conditions and loaded onto a 4–12 % sodium dodecyl sulfate-polyacrylamide gel (Thermo Fisher). 5–20 μg of protein was run through the gel at 120V for 1 h. The gel was blotted onto a PVDF membrane at 20V for 1 h using a ThermoFisher wet transfer system. The membranes were blocked using 5 % milk in Tris buffer saline Tween (TBST) buffer for 1–3 h. The membranes were washed with TBST and incubated with 5 % BSA in TBST containing the primary antibodies for the proteins of interest overnight in the fridge. Antibodies used: EphA2 (1:1000, Cell signalling, D4A2, Rabbit), phosphorylated-EphA2 Serine-897 (1:1000, Cell signalling, S897, Rabbit), alfa-Tubulin (1:1000, Cell signalling, 11H10, Rabbit). Next, the membranes were washed with TBST and incubated with the secondary antibody for 2–3 h at room temperature. Afterwards, the membranes were washed with TBST and signal was visualized using a SuperSignal™ West Pico Detection Kit for rabbit IgG that produces a chemiluminescent signal when exposed to UV light. Pictures were developed using a Uvitec Alliance developer.

### 2.10 Animal model

All animal experimentation procedures were conducted with approval by the Animal Ethical Committee of Erasmus University Medical Center (License number AVD101002015114, protocol number 16-691-06). 12-week-old male C57BL/6 mice (C57BL/6J0laHsd, 27.01 g ± 2.05 g; Envigo, Cambridgeshire, UK), were housed in groups of 8 in individually ventilated cages and maintained on a 12 h light/dark cycle with ad libitum access to standard diet and water at the Experimental Animal Facility of the Erasmus MC. Mice were randomly divided into two experimental groups (N = 8 per group): Control and ALW-II-41-27-treated mice. For all procedures, mice were anesthetized using 3 % isoflurane/0.8 L O_2_/min (Pharmachemie BV, Haarlem, the Netherlands). OA was induced unilaterally by intra-articular injections of 60 μg Monoiodoacetate (MIA) (Sigma-Aldrich, St. Louis, USA) in 6 μl of saline (0.9 % NaCl; Sigma-Aldrich) at day 0. Injections were performed after a 3–4 mm dermal incision was made to the right knee at the height of the patellar tendon. All intra-articular injections were administered using a 50 μl syringe (Hamilton, Bonaduz, Switzerland) and 30G needle (BD Medical, New Jersey, USA). ALW-II-41-27 was delivered using Alzet micro-osmotic pumps (Durect Corporation, CA, USA) model 1004, delivery rates of 0.11 μL/hour, that were implanted subcutaneously on the back of the mice, slightly posterior to the scapulae, immediately after the intra-articular injections. Osmotic pumps were filled with dimethyl sulfoxide: polyethylenglicol alone (55:45 ratio, vehicle-treated group, N = 8 mice) or containing 6 mg of ALW-II-41-27 dissolved in vehicle (treated group, N = 8 mice) which leads to a dose of 6.6 μg/hour. A third group of N = 8 mice received the implantation of osmotic pumps delivering a dose of 1.7 μg/hour of ALW-II-41-27. Mice were evaluated daily for signs of behavioral changes by the animal care staff at the experimental animal facility. Mice were euthanized in agreement with the Directive 2010/63/EU by cervical dislocation under isoflurane anesthesia 14 days following MIA injection. After which knees were fixed in 4 % formalin (v/v) for 1 week, decalcified in 10 % EDTA for 2 weeks and embedded in paraffin. Coronal sections of 6 μm were cut for analyses.

### 2.11 Flow cytometric analysis of peripheral blood monocytes

Peripheral blood was harvested from the facial vein of mice on days 2- and 12 post-induction of OA, as previously described [27]. Blood sampling order was performed randomly at each time-point. 50 μl of whole blood was pre-incubated with purified rat anti-mouse CD16/CD32 (BD Biosciences Cat# 553140, New Jersey, USA) for 5 min on ice. Blood was stained for the expression of CD11b (BioLegend, San Diego, USA, Cat# 101228), CD115 (BioLegend, Cat# 135505), Ly-6C (BioLegend Cat# 128005) and CD62L (BioLegend, Cat# 104412) to identify myeloid cells and specific monocyte subsets, as well as CD3 (BioLegend, Cat# 100220), NK1.1 (BioLegend, Cat# 108713), CD19 (BioLegend, Cat# 115520) and Ly-6G (BioLegend, Cat# 127618) to eliminate T cells, natural killer cells, B cells and neutrophils (Supplementary Table 3). Cells were stained for 30 min at 4 °C in the dark, followed by incubation with 2 mL of 1X FACS lysing solution (BD Biosciences) for 10 min to lyse red blood cells. Following centrifugation at 400*g* for 10 min, supernatant was removed, and cells washed and resuspended in FACSFlow buffer (BD Biosciences).

All samples were analyzed using a FACSJazz cytometer (BD Biosciences) and FlowJo software version 10.0.7 (FlowJo LLC, Oregon, USA). The gating strategies applied for blood monocytes analysis are presented in Supplementary Fig. 8.

### 2.12 Histological analyses of murine knee joints

To evaluate cartilage damage, sections were stained with Safranin O & Fast Green. To evaluate synovial inflammation and osteophyte area, sections were stained with Hematoxylin & Eosin. Images were acquired using the NanoZoomer Digital Pathology program (Hamamatsu Photonics, Ammersee, Germany). For each knee, 3 sections of the patellofemoral compartment, taken at standardized locations in the knee with 180 μm distance in between were evaluated by two independent well-trained evaluators that were blinded for the treatment (MNFB and NK). For each knee, the average value of the three sections was calculated and the values of two observers were averaged and used for representation and statistical analyses.

Cartilage damage was evaluated with the Osteoarthritis Research Society International (OARSI) scoring system [28].

Osteophyte size was assessed by measuring the area of the osteophyte at the lateral side of the patella, the location where the incidence of osteophytes was highest, using the NanoZoomer digital pathology program.

Synovial thickness was measured from the capsule to the superficial layer of the synovial membrane at the medial and lateral sides of the parapatellar recesses (three positions per section).

Synovitis was evaluated using the Krenn score [29] which considers three features of chronic synovitis (enlargement of lining cell layer, cellular density of synovial stroma, leukocytic infiltrate) and grade each feature from 0 (absent) to 3 (strong). The sum provided the synovitis score, which is interpreted as follows: 0–1, no synovitis; 2–4, low-grade synovitis; 5–9, high-grade synovitis.

To evaluate type X collagen in mice knees, a pre-coupling step was performed 24 h before the staining using a 1:10 dilution of collagen type X antibody (Quartett, #X53) with fluorescent goat anti-rat IgG (H + L) (Cross-Adsorbed Secondary Antibody, Alexa Fluor 546, #A11081, Fisher Scientific, Landsmeer, The Netherlands). Mouse IgG1 antibody (Dako Cytomation #X0931) dilution 1:10, was used as a negative control. Antigen retrieval was performed using Pepsin (Sigma #P7000) 1 mg/mL in 0.5M acetic acid pH2 for 2 h following 10 mg/mL hyaluronidase (Sigma #H3506) for 30 min. Slides were incubated with 10 % normal goat serum (Southern Biotech #0060-01) for 30 min. Slides were incubated overnight with the pre-coupled antibodies and following day slides were mounted using ProLong Diamond Antifade Mountant with DAPI (#P36966, Fisher Scientific, Landsmeer, The Netherlands).

To evaluate macrophages in the synovial membrane, F4/80 was used as a marker and a similar approach to type X collagen staining was performed using 1 μg/mL primary antibody F4/80 (eBioscience #14-4801-82, Waltham, MA, USA).

### 2.13 Pain measurement: hind limb weight distribution

Hind limb weight distribution was monitored as a surrogate pain indicator using an Incapacitance Tester (Linton Instrumentation, Norfolk, UK). Mice were positioned on the Incapacitance Tester with each hind limb resting on a separate force plate. Animals were habituated to the apparatus, three times per week, starting 2 weeks prior to the experiments. The examiner performing the measurements was blinded for treatment condition (MNFB). A baseline measurement was performed at day – 1, just before the induction of the OA. Follow up measurements were performed at day 1, 2, 3, 5, 7, 9 and 12. For data analyses, measurements with a registration below 10 g (<30 % of total body weight) in total on both hind limbs were excluded. 10 measurements were recorded per mouse per time point, of which at least 7 measurements were available on average. For each time point per mouse, the average of these measurements was used to calculate the percentage of weight on the affected limb as an indication of pain in the affected limb.

### 2.14 Data and statistical analyses

Statistical evaluation was performed using GraphPad Prism 9.0 and IBM SPSS 24 (IBM). Each *in vitro* experiment included at least 3 biological replicates and was repeated with cells derived from 3 donors. The *in vivo* study was designed to generate groups of equal size, used randomization and blinded analyses. The declared group size is the number of independent values that were used for statistical analysis. Sample size for the *in vivo* study was calculated considering the weight distribution over the hind limbs, as readout parameter. Based on previous studies, we consider an increase of 13 % (standard deviation of 10 %) in weight distribution on the affected limb in time in the therapy groups as relevant in our study [30]. Sample size was calculated with a statistical power of 80 % and significance level of 0.05, which led to N = 8. For statistical analysis, the linear mixed model with Bonferroni's multiple comparisons test was performed.

## 3 Results

### 3.1 *EPHA2* is as an inflammation-related gene upregulated in hypertrophic chondrocytes and osteoarthritic cartilage

**Fig. 1:**
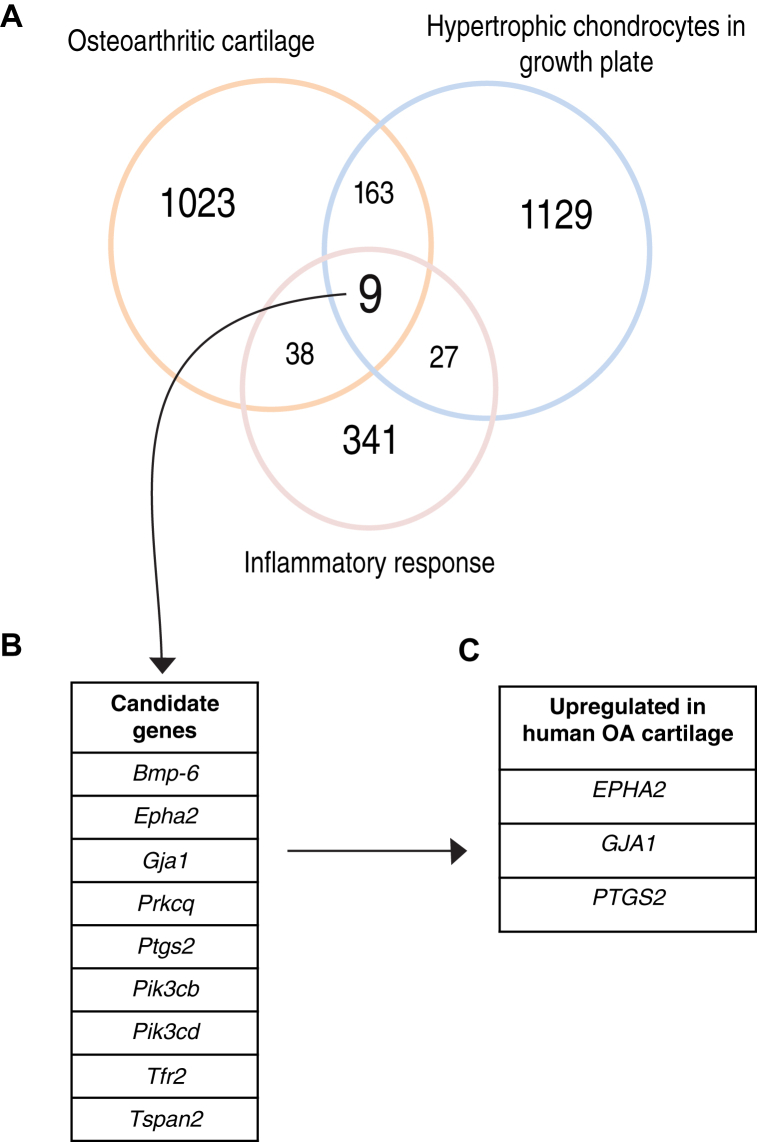
**Identification of EPHA2 as a novel target for OA associated with inflammation and chondrocyte hypertrophy**. (A) The Venn diagram illustrates the genes related to the inflammatory response, genes that were differentially regulated in murine osteoarthritic cartilage (DMM vs sham) and genes differentially expressed in the murine growth plate (proliferative vs hypertrophic zone). The number of genes in each dataset is represented, together with those that overlapped. (B) List of 9 targets that overlapped in the three databases. (C) Target genes upregulated in human OA compared with healthy articular cartilage. DEGs with a fold change greater than 3, and an adjusted p value lower than of 0.05, were considered for the analysis.

To identify a new therapeutic target for OA linked with chondrocyte hypertrophy and inflammation, we conducted data analysis on two publicly available murine microarray datasets [16,17]. Differentially expressed genes (DEGs) in the articular cartilage of mice with OA induced by destabilization of the medial meniscus (DMM) were compared to those from mice undergoing sham surgery, identifying OA-related genes. By intersecting this set with DEGs in the hypertrophic zone versus the proliferative zone of the mouse growth plate, we identified 172 genes differentially expressed in OA associated with chondrocyte hypertrophy. Among these, nine genes were associated with the gene ontology inflammatory response [19] (Fig. 1A and B). Subsequently, we examined the expression of these nine genes in a human microarray dataset obtained from OA and healthy articular cartilage [18], finding that three genes were also upregulated in human OA cartilage (Fig. 1 C). While *GJA1* and *PTGS2* have been previously studied in the context of OA [31], *EPHA2*, a tyrosine kinase receptor, has an undisclosed role in OA. *Epha2* was 30-fold upregulated in OA versus sham mouse articular cartilage (adjusted p-value = 2E-02) [17], and 3-fold upregulated in human OA versus healthy cartilage (adjusted p-value = 1E-08) [18]. Additionally, *Epha2* was 19-fold higher in the hypertrophic compared to the proliferative zone of the murine growth plate (adjusted p-value = 1E-07) [16].

**Fig. 2:**
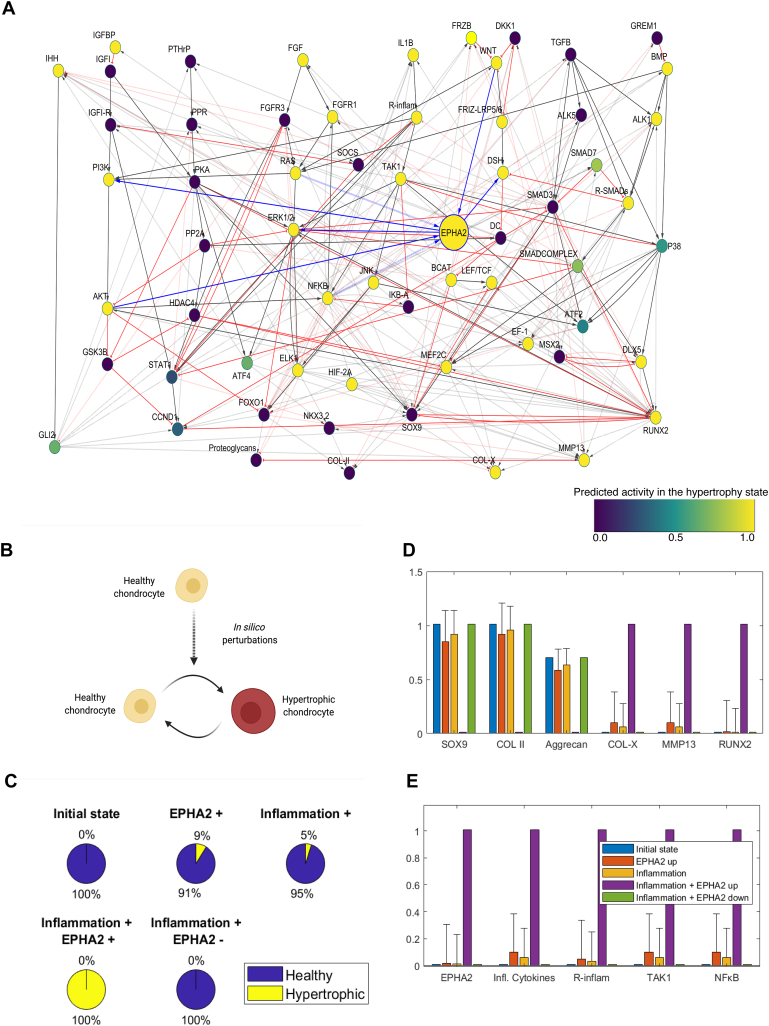
EPHA2 activation induces chondrocyte hypertrophy and inflammation *in silico* (A) Integration of EPHA2 in the regulatory network of articular chondrocytes. Solid lines denote protein interaction while modulation at the gene level is in dotted lines. Activating interactions are in grey with delta arrows and inhibitory interactions are in red with a half circle. Connections with EPHA2 are denoted in blue. All components of the model are represented with a variable that is named with capital letters. The variable represents neither the protein activation level nor the gene expression but a product of both (global activity). The color of the nodes in the network denotes the global activity of the variables in the hypertrophic state, with dark blue being 0 and yellow being 1. (B) Effect of gradually increasing the value of the EPHA2 input (from 0 to 1) in the scenario with EPHA2 activation and Inflammation, on the chondrocyte state transition. The initial state being the healthy (SOX9 +). (C) Activation of EPHA2 in the healthy state promotes the transition to a hypertrophic phenotype. Forced activation (+) or inhibition (−) of the entities from the initial healthy state is shown. Inflammation represents the activation of the variables related to inflammatory cytokines and their receptors, as input in the model. Pie charts represent the predicted percentage of perturbations leading to a transition to the hypertrophic phenotype or remaining in the healthy phenotype. (D) Average activity of the chondrogenic markers, being type II collagen, Aggrecan and SRY-box transcription factor (SOX9), and of hypertrophic markers, being Runt-related transcription factor 2 (RUNX2), Matrix Metallopeptidase (MMP13) and type 10 Collagen. (E) Average activity of EPHA2 and variables associated with inflammation. The bars represent the mean of the results of a hundred *in silico* experiments (+ standard deviations, +SD). There is no SD for the initial state (blue condition) as it denotes the starting point before the *in silico* perturbation is applied. Figure created with Cytoscape, MATLAB and Biorender.com.

### 3.2 EPHA2 triggers inflammatory signaling activation and hypertrophy in a virtual chondrocyte

To assess the role of EPHA2 on chondrocyte phenotype we utilized a computational model representing two cellular states: healthy and hypertrophic chondrocytes (Fig. 2 A) [22,32]. EPHA2 exhibited activity in the hypertrophic state but remained inactive in the healthy state (Supplementary Fig. 1). This activation refers not only to the transcription of the EPHA2 gene but also to its protein kinase activity. Activation of EPHA2 led to a more pronounced transition of healthy chondrocytes towards the inflammatory hypertrophic state compared to activation of inflammatory cytokines (9 % and 5 % for 'EPHA2+' and 'Inflammation+', respectively; Fig. 2B and C). Consequently, both conditions prompted a decline in anabolic markers (collagen type II, Aggrecan: blue vs orange and yellow bars; Fig. 2 D) and an increase in hypertrophic and inflammatory markers (Fig. 2D and E). Full activation of both EPHA2 and inflammatory cytokines synergistically induced a complete transition of healthy chondrocytes towards the inflammatory hypertrophic state ('Inflammation + EPHA2+'; Fig. 2 C). Intriguingly, inhibiting EPHA2 (Inflammation + EPHA2-) abolished this cellular state switching, accompanied by a significant reduction in the activity of hypertrophic and inflammatory entities (green vs violet and yellow bars; Fig. 2D and E). These computational findings substantiate the hypertrophic and inflammatory role of EPHA2 in chondrocytes.

### 3.3 ALW-II-41-27 reduces human OA-derived chondrocyte inflammation

**Fig. 3:**
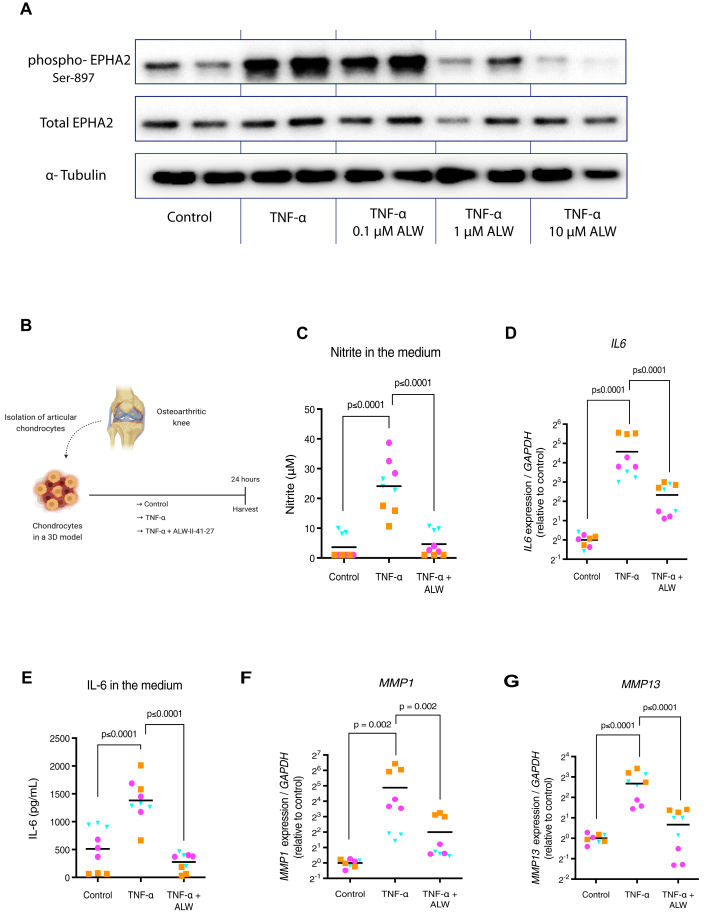
ALW-II-41-27 decreases TNF-*α* induced catabolism and inflammation in human chondrocytes. (A) ALW-II-41-27 decreases TNFα-induced phosphorylation of EPHA2 in a dose-dependent manner. (B) Experimental set-up to evaluate the anti-inflammatory capacity of ALW-II-41-27 (10 μM) in human OA chondrocytes cultured with 10 ng/mL TNF-α. (C) Evaluation of nitrite in the medium as a marker for inflammatory activity as determined by Griess reagent. (D, F-G) Gene expression of the inflammatory cytokine *IL6*, and the catabolic enzymes *MMP1* and *MMP13* determined by qPCR. The average of control, per donor, is set to 1. (E) IL-6 in the medium determined by ELISA. Experiments were performed in triplicate, with cells from three donors. Donors are represented with different colors and symbols: violet circles (donor 1), blue triangles (donor 2) and orange squares (donor 3). The horizontal line in the graphs represents the mean. Data were analyzed with the linear mixed model with Bonferroni's multiple comparisons test. Figure created with Biorender.com.

To investigate the potential of pharmacologically inhibiting EPHA2 to alleviate inflammation in OA, we utilized the tyrosine kinase inhibitor ALW-II-41-27, a type II kinase inhibitor known for its high selectivity for EPHA2 [33,34]. Initially, we validated the ability of ALW-II-41-27 to reduce TNF-α-induced phosphorylation of EPHA2 in a dose-dependent manner (Fig. 3 A). TNFα has been shown to activate EPHA2, inducing its phosphorylation at Serine 897 [14]. Notably, the concentration of 10 μM exhibited the highest efficacy in decreasing both EPHA2 phosphorylation and the expression of catabolic enzymes MMP1 and MMP13 in OA cartilage explants (Supplementary Fig. 4). Subsequently, we examined the effects of ALW-II-41-27 at a concentration of 10 μM on TNF-α-treated human chondrocytes from OA donors (Fig. 3 B). Treatment with ALW-II-41-27 significantly inhibited TNF-α-induced inflammatory responses, as evidenced by a reduction in the secretion of nitric oxide metabolites (Fig. 3 C). Furthermore, ALW-II-41-27 administration mitigated the TNF-α-induced expression of the inflammatory cytokine interleukin (IL)-6, known to be a downstream target of TNF-α (Fig. 2D and E) [35]. Moreover, ALW-II-41-27 treatment countered the TNF-α-induced upregulation of the cartilage matrix-degrading enzymes MMP1 and MMP13 (Fig. 3F and G). These findings demonstrate the anti-inflammatory potential of ALW-II-41-27 in TNF-α-stimulated OA chondrocytes.

### 3.4 ALW-II-41-27 decreases chondrocyte hypertrophy

**Fig. 4:**
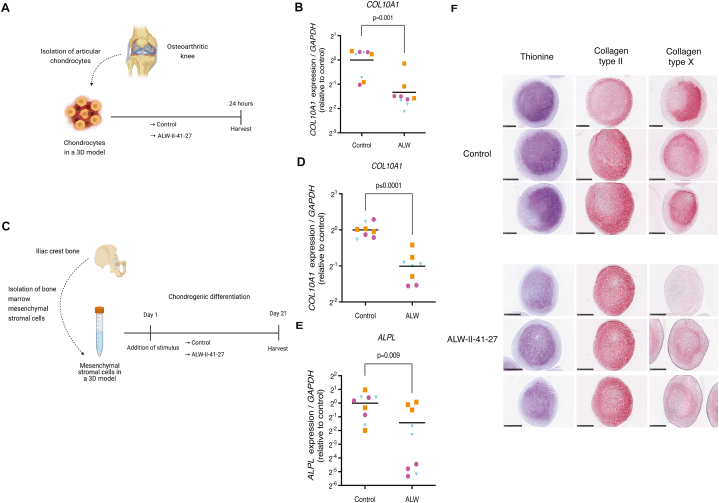
ALW-II-41-27 decreases chondrocyte hypertrophy. (A) Experimental set-up to evaluate the capacity of ALW-II-41-27 (10 μM) to modulate hypertrophy in human OA chondrocytes. (B) Gene expression of the hypertrophic marker *COL10A1* in cultured OA chondrocytes determined by qPCR. (C) Experimental set-up to evaluate how ALW-II-41-27 (100 nM) affects hypertrophy in tissue engineered cartilage from MSCs. (D, E) Gene expression of *COL10A1* and *ALPL* in MSC-generated cartilage determined by qPCR. (F) Histological analysis of tissue engineered cartilage derived from MSCs; Thionine staining showing glycosaminoglycans (violet), and immunohistochemistry of type II and type X collagen (red/pink). Representative images from three MSC-chondrogenic pellets per condition per staining are shown. Experiments were performed with 3 replicates, for each of the three donors. Donors are represented with different colors and symbols: violet circles (donor 1), blue triangles (donor 2) and orange squares (donor 3). For gene expression analysis, the average of control replicates is set to 1 per donor. The horizontal line in the graphs represents the mean. For statistical analysis, the linear mixed model with Bonferroni's multiple comparisons test was performed. Figure created with Biorender.com.

We then proceeded to evaluate whether ALW-II-41-27 could impede hypertrophy in human OA-derived articular chondrocytes (Fig. 4 A). ALW-II-41-27 decreased *COL10A1* expression (Fig. 4 B), suggesting a reduction in hypertrophy. Given that chondrocyte hypertrophy poses a significant obstacle to stable hyaline cartilage tissue engineering [36], we further investigated the effect of ALW-II-41-27 on cartilage tissue engineered constructs from mesenchymal stromal cells (MSCs), well-known to become hypertrophic and prone to ossify when implanted *in vivo* [[37], [38], [39], [40]] (Fig. 4 C). Gene expression analysis indicated that the addition of ALW-II-41-27 mitigated the hypertrophic markers *COL10A1* and *ALPL* (Fig. 4D and E). Likewise, when treated with ALW-II-41-27, the cells exhibited reduced deposition of type X Collagen (Fig. 4 F and Supplementary Fig. 5). Intriguingly, neither glycosaminoglycan nor type II Collagen deposition showed reduction, suggesting that ALW-II-41-27 primarily targeted hypertrophy.

### 3.5 ALW-II-41-27 treatment reduces joint inflammation and pathological endochondral ossification *in vivo*

**Fig. 5:**
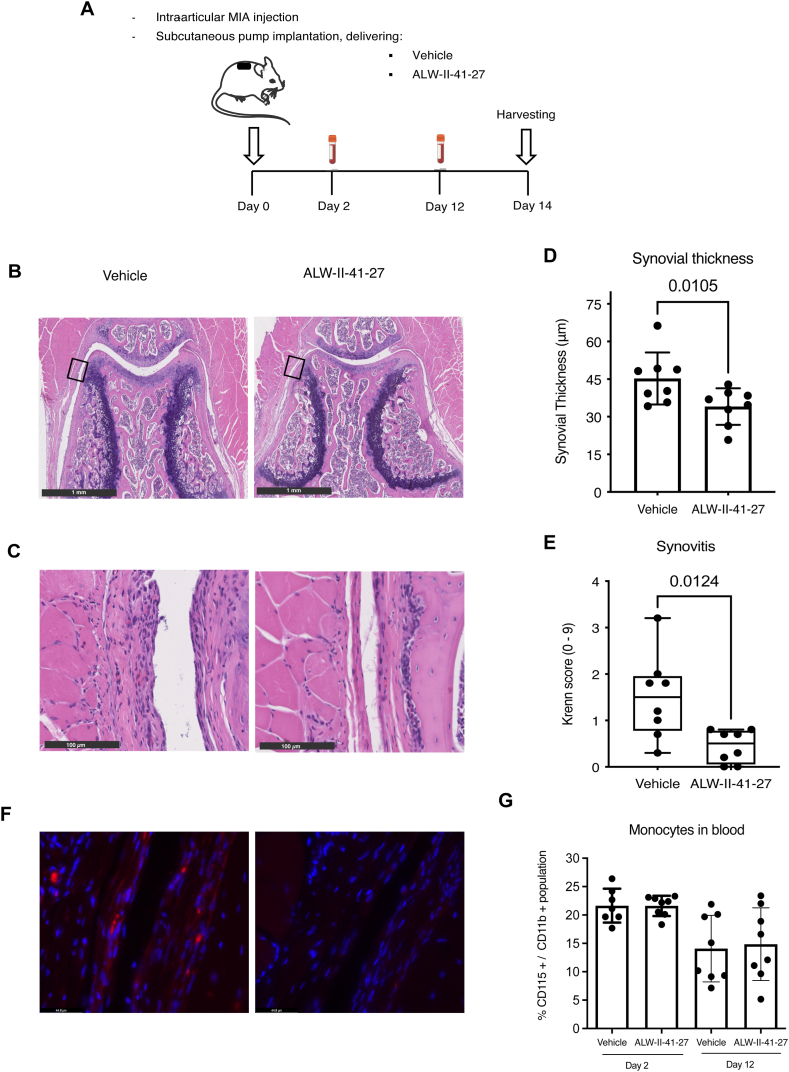
ALW-II-41-27 treatment attenuates joint inflammation. (A) Experimental set-up of the *in vivo* experiment. Intra-articular injection of monoiodoacetate (MIA, 60 μg in 6 μl of saline) was applied to the right knee of mice for each experimental group (N = 8) to induce OA. An osmotic pump was implanted on the back of the mice, slightly posterior to the scapulae, which continuously delivered vehicle or ALW-II-41-27 in a dose of 6.6 μg/hour. At day 2 and 12 peripheral blood was harvested. At day 14 mice were euthanized to assess the effects of ALW-II-41-27 on the degenerated joint. (B) Hematoxylin and Eosin staining of knees (patellofemoral region) from vehicle-treated and ALW-II-41-27-treated mice. Black square indicates the region of magnification for the image below (C) showing the synovial lining where synovial thickness and Krenn score were determined. (D) Synovial thickness is represented by the mean ± SD. (E) Krenn score illustrated with box-and-whiskers plots, with line indicating the median and error bars spanning maximum to minimum values. (F) Immunofluorescence of F4/80 (red) showing macrophages in the synovial lining, DAPI (blue). (G) Percentage of monocytes present in the peripheral blood of mice at day 2 and 12, respect to the myeloid cell population. In all graphs, each dot represents data of an individual mouse (N = 8). For statistical analysis, the linear mixed model with Bonferroni's multiple comparisons test was performed. Figure created with Biorender.com.

EPHA2 inhibition reduced hypertrophy and inflammation *in silico* and addition of the inhibitor ALW-II-41-27 in cell culture *in vitro* confirmed these results. Subsequently, we sought to assess the potential therapeutic efficacy of ALW-II-41-27 *in vivo* using a mouse model of joint pain and degeneration induced by intra-articular injection of monoiodoacetate (MIA) [41]. ALW-II-41-27 was administered subcutaneously with a controlled delivery system (Fig. 5 A). The mice showed no obvious changes in behavior and their total body weight remained unaffected by ALW-II-41-27 administration throughout the 14-day study period (Supplementary Fig. 6). While this suggests the drug did not cause noticeable effects on well-being during a treatment of 2 weeks, further investigation is required to fully assess its impact on the overall health of the animals, in particular when longer term treatment is applied. Notably, treatment with ALW-II-41-27 led to a reduction in synovial membrane thickness and synovitis compared to vehicle-treated mice (Fig. 5 B, C, D & E). Synovitis is governed by macrophages, the crucial regulators of OA progression and primary mediators of the inflammatory response [27,42]. Macrophages were predominantly observed in the synovial lining of vehicle-treated mice, while their presence appeared reduced in the ALW-treated group, suggesting a potential attenuation of joint inflammation (Fig. 5F). Notably, there were no discernible alterations in peripheral blood monocyte levels at day 2 and 12 (Fig. 5 G), indicating selective reduction of local joint inflammation without affecting systemic immune cells.

Despite joint pain being a significant OA symptom often associated with inflammation [43], no significant difference in weight distribution between limbs was detected between ALW-II-41-27-treated and vehicle-treated mice, suggesting no apparent effect on pain (Supplementary Fig. 7).

**Fig. 6:**
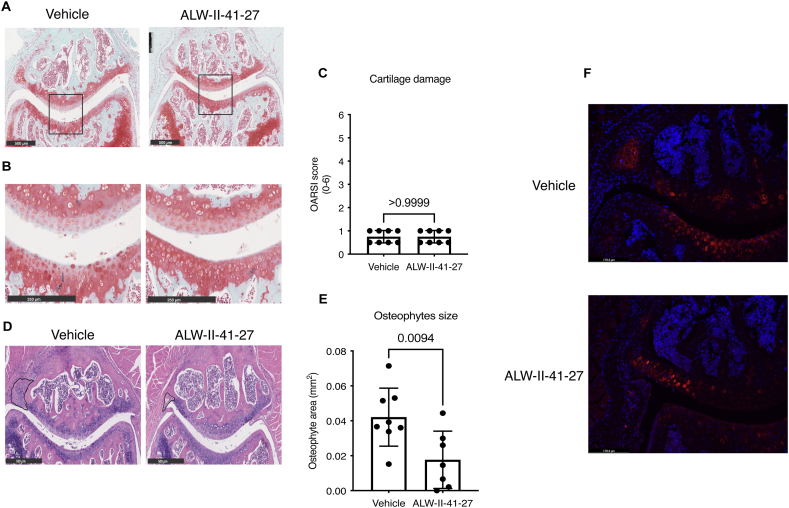
ALW-II-41-27 treatment attenuates pathological endochondral ossification (A) Safranin-O/Fast Green staining of vehicle-treated and ALW-II-41-27-treated mice knees with magnification of the patellofemoral region. Black square indicates the region of magnification for the image below (B) showing the central part of the patellofemoral articular cartilage. (C) OARSI score. Each dot represents data of an individual mouse (n = 8). (D) Hematoxylin and Eosin stain of vehicle-treated and ALW-II-41-27-treated mice knees with magnification of the patellofemoral region. Osteophyte's diameter is indicated with a black line in the lateral side of the patella. (E) Osteophyte area adjacent to the lateral side of the patella is represented by the mean ± SD. Each dot represents data of an individual mouse (n = 8). For statistical analysis, the linear mixed model with Bonferroni's multiple comparisons test was performed (F) Immunofluorescence of type X collagen (red) and DAPI (blue) in the lateral side of the patellofemoral region.

Cartilage degeneration, evidenced by proteoglycan loss, was observed in all mice irrespective of ALW-II-41-27 treatment (Fig. 6A and B & C), suggesting that ALW-II-41-27 was not able to rescue matrix degeneration *in vivo*. However, histological assessment revealed significantly smaller osteophytes, particularly at the lateral side of the patella, in ALW-II-41-27-treated mice (Fig. 6D and E). Additionally, type X collagen deposition was reduced in the knees of ALW-II-41-27-treated mice (Fig. 6 F), suggesting a potential attenuation of endochondral ossification, particularly visible in the osteophyte formation in the diseased joint.

## 4 Discussion

**Fig. 7:**
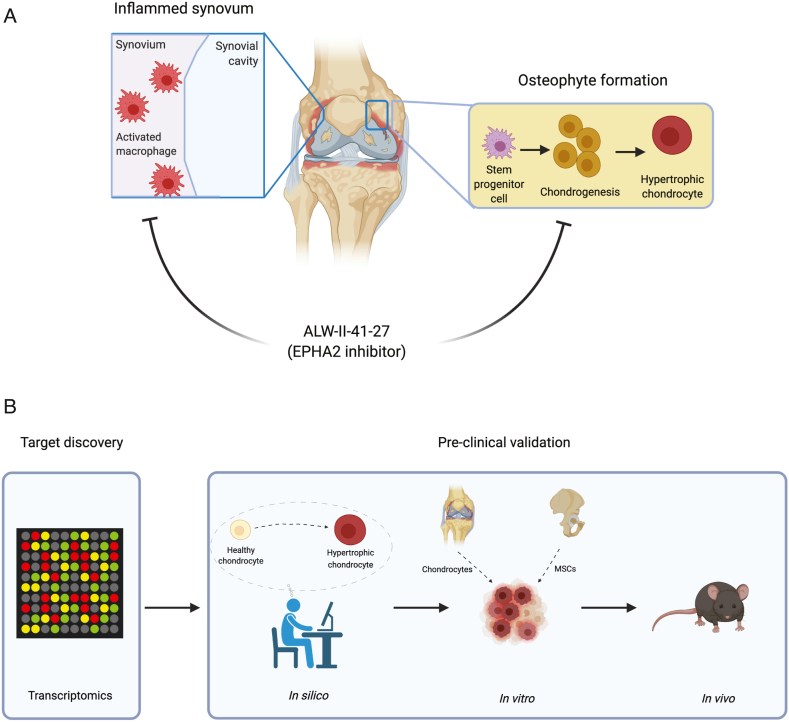
Graphical representation of main findings and experimental approach. (A) ALW-II-41-27 attenuates synovitis and osteophyte formation. (B) Drug discovery pipeline combining transcriptomic datasets, *in silico*, *in vitro* and *in vivo* models. Figure created with Biorender.com.

There is an urgent unmet need for effective therapies for OA patients. Using a multi-model approach we here show that EPHA2 is a promising drug target for OA and we report that the small molecule ALW-II-41-27 shows potency as a disease-modifying OA drug (DMOAD), specifically targeting inflammation and pathological endochondral ossification (Fig. 7A). To find targets associated with inflammation and chondrocyte hypertrophy we have used a particular sequence of studies that involved *in silico*, *in vitro* and *in vivo* analyses (Fig. 7 B). For the i*n silico* analyses we leveraged previously published large gene expression dataset depositories and narrowed them down to one target of interest. The role of the identified target on inflammation and chondrocyte hypertrophy was further investigated through *in silico* experiments using a computational model of cellular signaling networks controlling chondrocyte phenotypes. These *in silico* studies served as the starting point for a series of *in vitro* experiments using different cell models, which were followed by an *in vivo* study. Our study illustrates the efficacy of this experimental approach in uncovering a novel target for specific biological processes in osteoarthritis.

While previous research has implicated EPHA2 as a key player in diseases like cancer [[44], [45], [46], [47]] and irritable bowel disease [48], our study marks the first to underscore its significance in OA. Other tyrosine kinases, including fibroblast growth factor receptor (FGFR) 1, Fyn and vascular endothelial growth factor receptor (VEGFR), have been implicated to promote chondrocyte hypertrophy [[49], [50], [51], [52]]. Our findings reveal that the tyrosine kinase EPHA2 not only contributes to hypertrophy but also to inflammation, making it a compelling target for mitigating pathological mechanisms in OA. While we did not directly assess EPHA2 activation in chondrocytes under hypertrophic and inflammatory conditions, our data provide indirect evidence of its role; specifically, transcriptomic analysis reveals increased EPHA2 expression in hypertrophic chondrocytes, and Western blot analysis showed that TNFα stimulation enhances EPHA2 phosphorylation, suggesting a potential activation of this receptor during inflammation.

We have identified a promising compound, ALW-II-41-27, demonstrating potential as a disease-modifying osteoarthritic drug (DMOAD). To date, a wide variety of DMOADs targeting chondrocyte hypertrophy have been tested in experimental pre-clinical studies [[53], [54], [55]]. It is unclear, however, whether those agents have been subjected to further research. Regulatory agencies, such as the Food and Drug Administration (FDA) or the European Medicines Agency (EMA), have not yet approved any existing disease-modifying pharmacological intervention for OA [56]. Considering the pre-clinical data of ALW-II-41-27 to modulate OA pathogenesis and its extensive pharmacological analysis in other conditions [[44], [45], [46], [47], [48]], it is expected that clinical trials involving this compound may pose lower risks with a higher likelihood of success. Our study did not show adverse effects of systematic application of ALW-II-41-27 for two weeks on gross behavior or the body weight of the animal. However, further investigation is required to fully assess its impact on the overall health of the animals, in particular for longer term application. Additional, local application of the drug into the affected joint might be considered.

In addition to cartilage hypertrophy, inflammation of the synovium plays a crucial role in OA pathology [57]. Our results indicate that ALW-II-41-27 exhibits anti-inflammatory properties, aligning with its previously observed effects in a model of irritable bowel syndrome [48]. EPHA2 is not limited to chondrocytes but is also expressed in various other cell types found in the synovium, including fibroblasts, monocytes, and macrophages [12,58]. Thus, the observed anti-inflammatory mechanism in our *in vivo* setting may also be linked to the action of ALW-II-41-27 on these cell types.

Our study has certain limitations. Although our findings suggest that EPHA2 contributes to the pathogenesis of OA and ALW-41-27 shows higher affinity for EPHA2, it is possible that other kinases were also inhibited both *in vitro* and *in vivo*. Additionally, we have not demonstrated that ALW-II-41-27 reduces EPHA2 phosphorylation *in vivo*. Furthermore, treatment initiation coincided with OA induction in our research. This decision was influenced by the progressive nature of the disease, posing a challenge for a DMOAD to reverse extensive joint structural changes in end-stage OA. Hence, maximizing the pharmacological benefits of ALW-II-41-27 to reduce inflammation and osteophytosis might be more effective if administered during the earlier stages of the disease. Our study did not demonstrate that *in vivo* administration of ALW-II-41-27 prevented cartilage loss or pain in the MIA mouse model. Further investigation using alternative experimental animal models is warranted to determine whether ALW-II-41-27 specifically targets hypertrophy and inflammation, or if its effects extend to preventing cartilage degeneration and alleviating pain. Additionally, exploring the potential effects of ALW-II-41-27 on post-traumatic OA or other non-chemically induced forms of OA is essential.

In conclusion, we present a novel approach for therapeutic discovery that integrates *in silico* simulations, *in vitro* experiments with patient-derived cells, and *in vivo* studies using a mouse model. Using this pipeline, we demonstrated that ALW-II-41-27 shows promise in mitigating inflammation and pathological endochondral ossification, highlighting its potential as a candidate drug for modifying the course of osteoarthritis. These findings warrant further investigation to fully explore its therapeutic potential.

## CRediT authorship contribution statement

**Mauricio N. Ferrao Blanco:** Writing – review & editing, Writing – original draft, Visualization, Validation, Supervision, Resources, Project administration, Methodology, Investigation, Formal analysis, Data curation, Conceptualization. **Raphaelle Lesage:** Writing – review & editing, Writing – original draft, Validation, Software, Resources, Project administration, Methodology, Investigation, Formal analysis, Data curation, Conceptualization. **Nicole Kops:** Methodology, Investigation. **Niamh Fahy:** Methodology, Investigation. **Fjodor T. Bekedam:** Methodology, Investigation. **Athina Chavli:** Methodology, Investigation. **Yvonne M. Bastiaansen-Jenniskens:** Writing – review & editing, Formal analysis, Data curation. **Liesbet Geris:** Writing – review & editing, Supervision. **Mark G. Chambers:** Writing – review & editing, Supervision. **Andrew A. Pitsillides:** Writing – review & editing, Supervision. **Roberto Narcisi:** Writing – review & editing, Supervision, Writing – original draft. **Gerjo J.V.M. van Osch:** Writing – review & editing, Supervision, Funding acquisition, Conceptualization, Data curation, Formal analysis, Investigation, Methodology, Project administration, Writing – original draft.

## Ethical statement

Animal experiments were approved by the medical ethical committee of the Erasmus MC, protocol EMC 16-691-06. Human articular cartilage was obtained with the approval of Erasmus MC, protocol MEC-2004-322 and mesenchymal stromal cells MEC-2014-16.

## Funding

This study was financially supported by the European Union's Horizon 2020 research and innovation programme under the Marie Sklodowska-Curie grant agreement no. 721432 Carbon and the Reumafonds ReumaNederland grant number 18-1-202.

## Declaration of competing interest

The authors declare the following financial interests/personal relationships which may be considered as potential competing interests:Gerjo van Osch reports financial support was provided by European Union Horizon 2020. Gerjo van Osch reports financial support was provided by Reumafonds ReumaNederland. If there are other authors, they declare that they have no known competing financial interests or personal relationships that could have appeared to influence the work reported in this paper.
