# Supplementary material for: A multi-model approach identifies ALW-II-41-27 as a promising therapy for osteoarthritis-associated inflammation and endochondral ossification
Source: Heliyon. 2024 Dec 4;10(23):e40871. doi: 10.1016/j.heliyon.2024.e40871 (PMC11664402; doi:10.1016/j.heliyon.2024.e40871)
Supplement: Multimedia component 3 [file mmc3.pdf]

Protein signaling			
Source	Target	Sign	Reference
EPHA2	Dsh	1	2
EPHA2	ERK1/2	1	5
EPHA2	PI3K	1	6 and 7
AKT	EPHA2	1	4
WNT	EPHA2	1	1

Genetic regulation			
Source	Target	Sign	Reference
NFKB	EPHA2	1	3
RAS	EPHA2	1	8
