# Supplementary material for: A multi-model approach identifies ALW-II-41-27 as a promising therapy for osteoarthritis-associated inflammation and endochondral ossification
Source: Heliyon. 2024 Dec 4;10(23):e40871. doi: 10.1016/j.heliyon.2024.e40871 (PMC11664402; doi:10.1016/j.heliyon.2024.e40871)
Supplement: Multimedia component 4 [file mmc4.pdf]

Antibody	Clone	Fluorophore
Anti-mouse/human CD11b	M1/70	PerCp-Cy5.5
Anti-mouse CD115	AFS98	PE
Anti-mouse Ly6C	HK1.4	FITC
Anti-mouse CD62L	MEL-14	APC
Anti-mouse Ly6G	1A8	PE-Cy7
Anti-mouse CD3	17A2	PE-Cy7
Anti-mouse NK1.1	PK136	PE-Cy7
Anti-mouse CD19	6D5	PE-Cy7
Anti-mouse F4/80	BM8	FITC
Anti-mouse CD86	GL-1	PE-Cy7
Anti-mouse CD206	C068C2	APC
Anti-mouse CD163	TNKUPJ	PE
Anti-mouse CD31	390	APC
